# Sensitive LC-MS/MS Methods for Amphotericin B Analysis in Cerebrospinal Fluid, Plasma, Plasma Ultrafiltrate, and Urine: Application to Clinical Pharmacokinetics

**DOI:** 10.3389/fchem.2021.782131

**Published:** 2021-11-29

**Authors:** Leandro Francisco Pippa, Maria Paula Marques, Anna Christina Tojal da Silva, Fernando Crivelenti Vilar, Tissiana Marques de Haes, Benedito Antônio Lopes da Fonseca, Roberto Martinez, Eduardo Barbosa Coelho, Lauro Wichert-Ana, Vera Lucia Lanchote

**Affiliations:** ^1^ Department of Clinical Analyses, Toxicology and Food Science, School of Pharmaceutical Sciences of Ribeirão Preto, University of São Paulo, Ribeirão Preto, Brazil; ^2^ Division of Infectious Diseases, Department of Internal Medicine, School of Medicine of Ribeirão Preto, University of São Paulo, Ribeirão Preto, Brazil; ^3^ Department of Neurosciences and Behavioral Sciences, School of Medicine of Ribeirão Preto, University of São Paulo, Ribeirão Preto, Brazil; ^4^ Division of Nephrology, Department of Internal Medicine, School of Medicine of Ribeirão Preto, University of São Paulo, Ribeirão Preto, Brazil; ^5^ Division of Medical Images, Nuclear Medicine, Department of Medical, Imaging, Hematology and Oncology, School of Medicine of Ribeirão Preto, University of São Paulo, São Paulo, Brazil

**Keywords:** amphotericin B, plasma, unbound fraction, urine, cerebrospinal fluid, LC-MS/MS, neurocryptococcosis, pharmacokinetics

## Abstract

Neurocryptococcosis, a meningoencephalitis caused by *Cryptococcus* spp, is treated with amphotericin B (AmB) combined with fluconazole. The integrity of the brain-blood barrier and the composition of the cerebrospinal fluid (CSF) may change due to infectious and/or inflammatory diseases such as neurocryptococcosis allowing for the penetration of AmB into the central nervous system. The present study aimed to develop LC-MS/MS methods capable of quantifying AmB in CSF at any given time of the treatment in addition to plasma, plasma ultrafiltrate, with sensitivity compatible with the low concentrations of AmB reported in the CSF. The methods were successfully validated in the four matrices (25 μl, 5–1,000 ng ml^−1^ for plasma or urine; 100 μl, 0.625–250 ng ml^−1^ for plasma ultrafiltrate; 100 μl, 0.1–250 ng ml^−1^ for CSF) using protein precipitation. The methods were applied to investigate the pharmacokinetics of AmB following infusions of 100 mg every 24 h for 16 days administered as a lipid complex throughout the treatment of a neurocryptococcosis male patient. The methods allowed for a detailed description of the pharmacokinetic parameters in the assessed patient in the beginning (4th day) and end of the treatment with AmB (16th day), with total clearances of 7.21 and 4.25 L h^−1^, hepatic clearances of 7.15 and 4.22 L h^−1^, volumes of distribution of 302.94 and 206.89 L, and unbound fractions in plasma ranging from 2.26 to 3.25%. AmB was quantified in two CSF samples collected throughout the treatment with concentrations of 12.26 and 18.45 ng ml^−1^ on the 8th and 15th days of the treatment, respectively. The total concentration of AmB in plasma was 31 and 20 times higher than in CSF. The unbound concentration in plasma accounted for 77 and 44% of the respective concentrations in CSF. In conclusion, the present study described the most complete and sensitive method for AmB analysis in plasma, plasma ultrafiltrate, urine, and CSF applied to a clinical pharmacokinetic study following the administration of the drug as a lipid complex in one patient with neurocryptococcosis. The method can be applied to investigate the pharmacokinetics of AmB in CSF at any given time of the treatment.

## 1 Introduction

Neurocryptococcosis is a subacute meningoencephalitis caused by the inhalation of the fungus *Cryptococcus* spp. After a latency period in the pulmonary lymph nodes, it spreads throughout the body with tropism for the central nervous system (Coelho et al., 2014). Neurocryptococcosis is more prevalent in HIV and other immunosuppressed patients and less commonly in individuals considered immunocompetent (Jarvis et al., 2010; Durski et al., 2013; Pyrgos et al., 2013; Rolfes et al., 2015; Williamson et al., 2016).

The treatment for neurocryptococcosis aims to sterilize the central nervous system and reduce intracranial pressure to values below 200 mmH_2_O considering that pressures above 350 mmH_2_O are associated with papilledema, decreased visual acuity, decreased hearing capacity, headaches, and confusion. Symptoms resulting from increased cerebrospinal fluid (CSF) pressure can be controlled by lumbar punctures to reduce pressure to levels below 200 mmH_2_O (Redmond et al., 2007).

Amphotericin B (AmB; C_47_H_73_NO_17_) is a polyene derived from *Streptomyces nodosus*, a compound discovered in the 1950s that remains the first line of treatment for invasive fungal infections, although new triazole antifungal drugs with a broad spectrum of action and good distribution to the central nervous system, such as voriconazole and posaconazole are also available (Black and Baden, 2007; Lestner et al., 2010).

AmB is mainly eliminated unchanged via biliary secretion. There are no known AmB metabolites described either in preclinical or clinical studies. Tissue accumulation accounts for most of the drug’s disposition. AmB can still be detected in the liver, spleen, and kidneys for up to 1 year after the end of therapy (Benson and Nahata, 1988). Based on experimental studies in mice, AmB is probably a substrate of P-glycoprotein (P-gp) (Wu et al., 2015) and breast cancer resistance protein (BCRP) (Stevens et al., 2015) drug transporters expressed in the brain-blood barrier (Cordon-Cardo et al., 1989; Schinkel et al., 1996).

The integrity of the brain-blood barrier and the composition of the CSF may change due to infectious and/or inflammatory diseases such as neurocryptococcosis (Shawahna, 2015) due to the high levels of pro-inflammatory cytokines and other inflammatory mediators (Williamson et al., 2016). *In vitro* studies indicate that the permeability of the brain-blood barrier for AmB is altered by TNF-α and lipopolysaccharide (Pyrgos et al., 2010). Considering that AmB is a Class 4 compound according to the Biopharmaceutics Drug Disposition Classification System (Shugarts and Benet, 2009; Hosey et al., 2016) and that central nervous system efflux and biliary secretion drug transporters play a significant role in the pharmacokinetics of this drug, adjustments to AmB dose may be necessary for neurocryptococcosis patients over the treatment evolution.

The first method for quantifying AmB in plasma dates from the late 1970s and reports the use of high-performance liquid chromatography (HPLC) with detection by UV or fluorescence (Nilsson-Ehle, 1977). Other methods applying HPLC or UHPLC (ultra-high-performance liquid chromatography) with detection by UV or fluorescence continue to be used over the years to date (Liu et al., 1995; Campanero et al., 1997; Echevarría et al., 1998; Groll et al., 2000; Lee et al., 2001; Wurthwein et al., 2005; Qu et al., 2017; Wirth et al., 2018).

The application of mass spectrometry (LC-MS/MS) to quantify was described for the first time by Lee et al. (Lee et al., 2001) in the assessment of four diffAmB erent biological matrices using 50 μl of plasma, urine, and human fecal homogenate with lowest limits of quantification (LLOQ) of 2000, 50 and 40 ng ml^−1^, respectively, and 200 μl of human plasma ultrafiltrate with an LLOQ of 1 ng ml^−1^. Other previous methods that apply LC-MS/MS (Lee et al., 2001; Deshpande et al., 2010; Strenger et al., 2014) have used larger volumes of biological matrices. In addition, these cited studies used the solid-phase extraction technique in the sample preparation, which makes the analysis of a large number of samples more expensive, a common scenario in clinical pharmacokinetics studies. LC-MS/MS was first used to analyze human CSF samples by Xiong et al. (2009), with 100 μl samples and an LLOQ of 0.5 ng ml^−1^.

The present study aimed to develop and validate LC-MS/MS methods for quantifying AmB total concentrations in plasma, urine, and CSF, as well as the unbound concentration in plasma using plasma ultrafiltrate. The investigation of the pharmacokinetics of AmB in patients with neurocryptococcosis requires the development and validation of methods with sensitivity compatible with the low concentrations of AmB reported in the CSF, taking into consideration the low volumes available for this matrix (Leenders et al., 1997; Wurthwein et al., 2005; Xiong et al., 2009; Hamill et al., 2010; Strenger et al., 2014; Stone et al., 2016). The methods described in the present study were successfully applied to quantify AmB in all available biological fluids including CSF samples from one patient with neurocryptococcosis up to the 16th day of treatment with AmB lipid complex.

## 2 Materials and Methods

### 2.1 Standard Solutions and Reagents

A stock solution of AmB (88.6%, European Pharmacopeia Reference Standard, CRS, Strasbourg, France) was prepared in methanol:dimethylsulfoxide (1:1, v/v) at the concentration of 1 mg ml^−1^. It was further diluted in methanol to obtain a concentration of 100 μg ml^−1^. This solution was used to prepare the working solutions in methanol at 0.4–1,000 ng ml^−1^. Piroxicam (C_15_H_13_N_3_O_4_S) (European Pharmacopeia Reference Standard, CRS, Strasbourg, France) used as the internal standard (IS) was prepared at the concentration of 1 mg ml^−1^ of methanol and diluted to concentrations of 10 and 100 ng ml^−1^ of methanol.

Since AmB is photosensitive (Beggs, 1978), all experiments were carried out under yellow light (sodium vapor lamps) as the only illumination source. Standard solutions were stored in BD Falcon^®^ polypropylene tubes (BD, Franklin Lakes, NJ, United States), aliquoted in polypropylene microtubes, protected by aluminum foil, and stored at −20°C.

Acetonitrile, methanol (J. T. Baker, Phillipsburg, NJ, United States), isopropanol (Honeywell Riedel-de Haën^®^, Seelze, Germany), and dimethylsulfoxide (Merck, Darmstadt, Germany) were used at chromatographic grade. Formic acid (J. T. Baker, 90.1%) and ammonium acetate (J. T. Baker, 98.9%) were used at analytical grade. Water was obtained from the Milli Q Plus^®^ purification system (Millipore, Bedford, MA, United States).

### 2.2 Chromatographic Analysis

AmB analysis was performed by liquid chromatography coupled to a tandem mass spectrometer (LC-MS/MS) consisting of a quaternary ACQUITY UPLC^®^ H-Class pump, ACQUITY UPLC^®^ Sample Manager—FTN automatic injector equipped with an ACQUITY Sample Organizer, TCM/CHM^®^ column oven, and XEVO TQ-S^®^ triple quadrupole mass spectrometer equipped with Zspray™ Electrospray Interface (ESI), all Waters Corp. (Milford, MA, United States).

The chromatographic analysis was conducted on a reverse-phase column LiChrospher^®^ 60 RP-Select B 5 µm particles, 125 × 4.6 mm, protected by a guard column LiChrospher^®^ 60 RP-Select B, 5 µm particles, 4 × 4 mm and kept at 25°C, all Merck (Darmstadt, Germany). The mobile phase consisted of an isocratic solvent system of 0.1% formic acid in water and 0.1% formic acid in acetonitrile in a ratio of 40:60 (v/v) for plasma or urine analysis and 50:50 (v/v) for plasma ultrafiltrate or CSF analysis, all at a flow rate of 0.8 ml min^−1^. The wash solution was prepared with 0.25% formic acid in 50:20:15:15 (v/v/v/v) acetonitrile:isopropanol:methanol:water solution.

### 2.3 Mass Spectrometry

MS/MS analysis was performed in positive ionization mode. The capillary voltage at the ESI was set to 3.50 kV. The source and desolvation temperatures were kept at 120 and 400°C, respectively. Nitrogen was used as a nebulization gas at a flow rate of 600 L h^−1^. Argon was used as the collision gas at a flow rate of 0.18 ml min^−1^. The cone energy was 30 V for both AmB and IS. The collision energies were 20 eV for AmB and 30 eV for IS.

The MS/MS conditions were optimized by the direct infusions of AmB and IS solutions at the concentration of 100 ng ml^−1^ prepared in a mixture of 0.1% formic acid in water and 0.1% formic acid in acetonitrile (1:1, v/v). The analysis was performed in the selected reaction monitoring mode. The protonated ions [M + H]^+^ and their respective product ions were monitored in the transitions of m/z 906 → 743 for AmB for all the four matrices and m/z 332 → 95 for IS in plasma and urine analysis, and m/z 332 → 121 in plasma ultrafiltrate and CSF analysis. Data acquisition and sample quantification were performed using MassLynx^®^ version 4.1 (Micromass, Manchester, United Kingdom).

### 2.4 Sample Preparation

#### 2.4.1 Total Plasma and Urine Samples

Aliquots of 25 μl of plasma or urine were prepared by adding 25 μl of IS solution in methanol (piroxicam, 100 ng ml^−1^), 25 μl of methanol, and further precipitated with 100 μl of the solution of 0.1% formic acid in acetonitrile. The tubes were shaken for 5 seconds and then centrifuged at 4°C for 15 min at 21,500 × *g* (refrigerated Himac CT15RE ultracentrifuge; Hitachi, Tokyo, Japan). Then, 100 μl of the supernatant were transferred to the injection vials and mixed with 100 μl of 0.1% formic acid in water, of which 20 μl of the final mixture were subjected to chromatographic analysis. The DQCs samples were diluted with the respective blank matrix in the proportion 1:4 (v/v) before the sample preparation process. Aliquots of 25 μl of the diluted DQC samples were transferred to a new microtube and processed following the same sample preparation procedure as a regular sample. Calibration curves were prepared similarly, enriching 25 μl of blank matrix (plasma or urine) with 25 μl of each working solution of AmB in methanol instead of 25 μl of methanol.

#### 2.4.2 Plasma Ultrafiltrate Samples

Total plasma was ultrafiltrated immediately before the sample preparation processes. Aliquots of 500 μl of plasma were added to Centrifree^®^ Ultrafiltration Devices (Merck, Darmstadt, Germany) and centrifuged at 37°C for 30 min at 1875 × *g* in a fixed angle rotor centrifuge at an angle of 36° (Novatecnica, model NT 875), according to the manufacturer’s indication. Aliquots of 100 μl of the obtained ultrafiltrate were processed by adding 25 μl of IS solution in methanol (100 ng ml^−1^), 25 μl of methanol and further precipitated with 50 μl of the solution of 0.1% formic acid in acetonitrile. The tubes were shaken for 30 s and then centrifuged at 4°C for 15 min at 21,500 × *g*. An aliquot of 100 μl of the supernatant was transferred to the injection vials, mixed with 100 μl of 0.1% formic acid in water, and 30 μl of the final mixture was chromatographed. The DQCs samples were diluted blank plasma ultrafiltrate in the proportion 1:10 (v/v) before the sample preparation process. Aliquots of 100 μl of the diluted DQC samples were transferred to a new microtube and processed following the same sample preparation procedure as a regular sample. Calibration curves were also prepared similarly, enriching 100 μl of blank plasma ultrafiltrate with 25 μl of each working solution of AmB in methanol instead of 25 μl of methanol.

#### 2.4.3 Cerebrospinal Fluid Samples

Aliquots of 100 μl of CSF at approximately 4°C were transferred to microtubes containing 25 μl of IS solution in methanol (piroxicam, 10 ng ml^−1^), 25 μl of methanol, and further precipitated with 50 μl of the solution of 0.1% formic acid in acetonitrile. The tubes were shaken for 30 s and then centrifuged at 4°C for 15 min at 21,500 × *g*. Then, 100 μl of the supernatant were transferred to the injection vials, and 50 μl of 0.1% formic acid in water were added, of which 20 μl of the final mixture were chromatographed. Calibration curves were also prepared similarly, enriching 100 μl of blank CSF with 25 μl of each working solution of AmB in methanol instead of 25 μl of methanol.

### 2.5 Method Validation

The analytical methods were developed and validated according to the Guideline on bioanalytical method validation of The European Medicines (European Medicines Agency, 2011). Blank plasma and urine samples were obtained from healthy volunteers after signing the prior informed consent form, and cerebrospinal fluid samples were provided by the Cerebrospinal Fluid Laboratory of the General Hospital of the Ribeirao Preto Medical School, University of São Paulo, according to the research project approved by the Research Ethics Committee of the School of Pharmaceutical Sciences of Ribeirão Preto, University of São Paulo and the Research Ethics Committee of the General Hospital of the Ribeirao Preto Medical School, University of São Paulo (Section 2.6 Clinical protocol).

#### 2.5.1 Calibration Curves

Calibration curves for the analysis of plasma or urine concentrations were prepared in triplicates using aliquots of a blank matrix (25 μl of plasma, 25 μl of urine, 100 μl of plasma ultrafiltrate, or 100 μl of CSF) enriched with 25 μl of each working standard solutions of AmB to obtain different AmB concentrations in the ranges of 5–1,000 ng ml^−1^ for plasma and urine, 0.625–250 ng ml^−1^ for plasma ultrafiltrate, or 0.1–250 ng ml^−1^ for CSF. Blank, zero, and enriched samples were submitted to the process described in items 2.4.1 for plasma or urine, 2.4.2 for plasma ultrafiltrate, and 2.4.3 for CSF.

Linear regression equations were obtained for each matrix by plotting the ratios of the AmB/IS areas as a function of their respective concentrations. Calibration standards must present deviations less than or equal to 20% to the nominal value in the LLOQQC concentration and deviations less than or equal to 15% to the nominal value for the other concentrations. Calibration curves are accepted when at least 75% of the calibration standards meet these requirements, and they must include the concentrations of the LLOQ and ULOQ.

#### 2.5.2 Quality Controls

Quality control (QC) solutions containing AmB were prepared in the respective biological matrix by adding the required volume of AmB standard in methanol, evaporating it under nitrogen flow, adding the necessary biological matrix and vigorously shaking (Phoenix Luferco solution shaker, model AP56, Araraquara, SP, Brazil) the solution for 3 min. The QC solutions for plasma and urine were prepared at final concentrations of 5 ng ml^−1^ (lowest limit of quantification quality control, LLOQQC); 10 ng ml^−1^ (low concentration quality control, LCQC); 500 ng ml^−1^ (medium concentration quality control, MCQC); 800 ng ml^−1^ high concentration quality control, HCQC), and 2,000 ng ml^−1^ (dilution quality Control, DQC). The QC solutions for the quantifications in plasma ultrafiltrate were prepared at the concentrations 0.625 ng ml^−1^ (LLOQQC), 1.5625 ng ml^−1^ (LCQC), 125 ng ml^−1^ (MCQC), 200 ng ml^−1^ (HCQC), and 500 ng ml^−1^ (DQC).

#### 2.5.3 Selectivity

The selectivities of the methods of analysis of AmB in plasma, plasma ultrafiltrate, urine, and CSF were evaluated in their respective blank matrices, eight for plasma and plasma ultrafiltrate (four normal, two hemolyzed, and two lipemic), six for urine (five normal and one obtained from a woman in the menstrual period) and eight for CSF (four normal, two yellowish-turbid, and two containing blood). The interfering peak areas at the same retention time of the AmB must be less than 20% of the area of the LLOQQC. The interfering peak areas near the IS retention time must be less than 5% of the IS area.

#### 2.5.4 Carryover Effect

The carryover effects of the methods were evaluated by performing three injections of each processed blank sample (plasma, plasma ultrafiltrate, urine, and CSF), one before and two after the injection of the respective matrix in the concentration of the upper limit of quantitation (ULOQ, 1,000 ng ml^−1^ of plasma or urine; 250 ng ml^−1^ of plasma ultrafiltrate or CSF). The interfering peak areas at the same retention time of AmB must be less than 20% of the area originating from the LLOQQC. The interfering peak areas at the IS retention time should be less than 5% of the IS area.

#### 2.5.5 Matrix Effect

The matrix effect (ME) was assessed at low and high concentration quality controls (LCQC and HCQC) levels in all three matrices. ME was assessed from eight plasma and plasma ultrafiltrate sources (four normal, two hemolyzed, and two lipemic), six urine sources (five normal and one from a woman in the menstrual period), eight CSF sources (four normal, two yellowish-turbid, and two containing blood). The samples were precipitated as described in items 2.4.1, 2.4.2, and 2.4.3, followed by the addition of the AmB and IS solutions to obtain the same concentrations as the LCQC and HCQC. The Matrix Factor Normalized by Internal Standard (MFNIS) was evaluated for each sample applying the equation below. The Coefficient of Variation (CV) for the group of samples from the same matrix must be less than 15%.

$$\mathrm{MFNIS=}\frac{\left(\mathrm{analyte area in matrix}\right)/\left(\mathrm{internal standard area in matrix}\right)}{\left(\mathrm{analyte area in solution}\right)/\left(\mathrm{internal standard area in solution}\right)}$$

MFNIS: Matrix Factor Normalized by Internal Standard.

Additionally, the matrix effect was also assessed by the classic post-column infusion test, injecting a processed sample from each biological matrix (plasma, urine, and CSF) with a combined infusion of the standard solution of AmB or the IS piroxicam, both at a concentration of 250 ng/ml and at a flow rate of 5 μl min^−1^. The same test was also performed by replacing the matrix (plasma, urine, and CSF) with water.

#### 2.5.6 Precision and Accuracy

The precision and accuracy of the methods were assessed through within-run and between-run assays. The assays were performed at the concentrations of LLOQQC, LCQC, MCQC, and HCQC for each matrix (plasma, urine, plasma ultrafiltrate, and CSF) and DQC for the methods of plasma, urine, and plasma ultrafiltrate.

In order to assess within-run precision and accuracy, five replicates of each concentration (LLOQQC, LCQC, MCQC, HCQC, and DQC) in plasma, plasma ultrafiltrate, urine, and CSF were analyzed in a single analytical run. For assessing the precision and accuracy of the between-run assays, five aliquots of each concentration in the four matrices were analyzed in three different analytical runs.

The assessment of within-run and between-run precision was performed by calculating the CV of the results obtained. To be accepted as accurate, the CVs must be equal to or less than 15% for all the concentrations, except for the LLOQQC with an accepted CV of 20%. The accuracy is expressed by the Relative Error (RE, inaccuracy), with accepted values within the range of ±15% of the nominal value, except for the LLOQQC, with accepted values within the range of ±20% of the nominal value.

$$\mathrm{RE=}\frac{\left(\mathrm{mean experimental concentration-nominal concentration}\right)}{\left(\mathrm{nominal concentration}\right)}\mathrm{\times 100}$$

RE: relative error (inaccuracy), %.

#### 2.5.7 Stabilities

AmB stability assays were conducted using five replicate samples at concentrations of LCQC (10 ng ml^−1^ for plasma or urine, 1.5625 ng ml^−1^ for plasma ultrafiltrate, and 0.25 ng ml^−1^ for CSF) and HCQC (800 ng ml^−1^ for plasma or urine, and 200 ng ml^−1^ for plasma ultrafiltrate and CSF). Freeze and thaw stability was assessed by freezing samples at −70°C for at least 12 h and thawing at 25°C for 60 min. After three cycles of freezing and thawing, samples were analyzed using freshly prepared calibration curves. Freeze and thaw stability was not assessed for plasma ultrafiltrate since all samples were analyzed immediately after the ultracentrifugation process. Short-term stability was assessed after keeping the samples at 25°C for 4 h for plasma and urine, at 25°C for 2 h for plasma ultrafiltrate, and at 4°C for 2 h for CSF. All samples were analyzed using freshly prepared calibration curves. Post-processing stability was assessed after the LCQC and HCQC samples were kept for 24 h at the auto-injector temperature of 15°C for plasma, urine, and CSF, or for 20 h at 12°C for plasma ultrafiltrate. All samples were analyzed using freshly prepared calibration curves. The stability of AmB in biological matrices is accepted when the deviation from the nominal value is equal to or less than ±15%.

#### 2.5.8 Non-Specific Binding to Ultrafiltration Membrane

Solutions of AmB were prepared in water in the same concentrations of the LCQC and HCQC of the ultrafiltrate QCs, respectively 1.5625 ng ml^−1^ and 200 ng ml^−1^. Aliquots of 500 μl of each solution were added to Centrifree^®^ Ultrafiltration Devices and centrifuged at 37°C for 30 min at 1875 × *g,* following the sample preparation protocol for plasma ultrafiltrate samples (2.4.2). Aliquots of 100 μl of both the obtained filtered solution or the unfiltered solution were prepared by adding 25 μl of the IS solution in methanol (piroxicam 100 ng ml^−1^), 25 μl of methanol, 50 μl of the solution of 0.1% formic acid in acetonitrile and were subsequently transferred to the injection vials, of which 30 μl of the final mixture were chromatographed. Calibration curves were also prepared similarly, enriching 100 μl water with 25 μl of each working solution of AmB in methanol instead of 25 μl of methanol. The non-specific binding to the ultrafiltration membrane was assessed by quantifying five replicates of the filtered and unfiltered solutions prepared in both concentrations. CV and RE were calculated for all samples of each concentration, with accepted values within the range of ±15% of the nominal value.

### 2.6 Clinical Protocol

**FIGURE 1:**
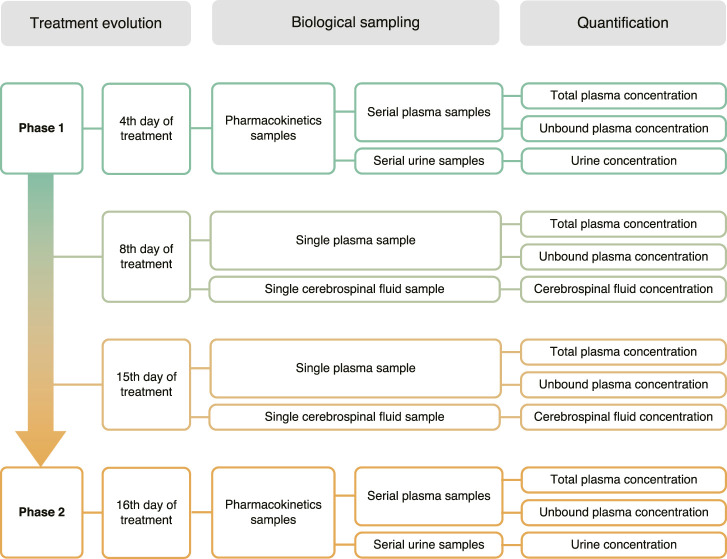
Clinical protocol and biological sampling strategy.

A 35-year-old white male admitted to the General Hospital of the Ribeirão Preto Medical School already under tuberculosis treatment was diagnosed with neurocryptococcosis. The subject was enrolled in this study previously approved by the Research Ethics Committee of the School of Pharmaceutical Sciences of Ribeirão Preto, University of São Paulo (CAAE: 96780618.7.0000.5403) and the Research Ethics Committee of the General Hospital of the Ribeirao Preto Medical School, University of São Paulo (CAEE: 96780618.7.3001.5440). As part of the treatment for neurocryptococcosis, the patient received daily doses of amphotericin B lipid complex (Abelcet^®^ 100, Teva Pharmaceutical Industries Ltd., Brazil) and fluconazole. The subject was assessed at the beginning (Phase 1, 4th day) and the end (Phase 2, 16th day) of the treatment with AmB. In both phases, the patient was requested to empty his bladder and then received 100 mg in 500 ml saline in a four-hour i. v. infusion. The complete list of medications in use in both phases is described in Table 1. The sampling protocol is shown in Figure 1. Serial blood samples (4 ml) were collected in EDTA tubes immediately before AmB infusion and after 2, 4, 5, 6, 7, 10, 14, 18, 22, 23, and 24 h. Blood samples were centrifuged at 4°C for 15 min at 1875 × *g*. The plasma was transferred to polypropylene cryogenic tubes and stored at −80°C. Urine was collected in individual flasks at every spontaneous micturition, the volume was measured, and an aliquot of 5 ml was stored at −80°C. CSF samples were obtained from the exceeding volume of regular lumbar punctures performed by the medical team for fungal culture tests or to relieve increased intracranial pressure. CSF samples were collected in tubes containing no additives and stored at −80°C. A blood sample was collected immediately with each CSF sample in an EDTA tube, processed and stored as described above for plasma samples.

**TABLE 1:** Anthropometric, biochemical, and hematological parameters of a male patient treated for neurocryptococcosis with a dosing regimen of 100 mg of amphotericin B lipid complex.

	References	Pretreatment	Phase 1	Phase 2
Age (years)		35		
Height (m)		1.75		
Weight (kg)		—	54.15	56.3
Amphotericin B dose (mg day^−1^)			100	100
Amphotericin B dose (mg kg^−1^ day^−1^)			1.85	1.78
Body mass index (kg m^−2^)	18.50–24.90	—	17.68	18.38
Creatinine (mg dl^−1^)	0.74–1.35	0.87	0.84	—
Urea (mg dl^−1^)	5.00–20.00	29.29	43.87	33.95
Alkaline phosphatase (U L^−1^)	44.00–147.00	115.7	163.18	—
Alanine aminotransferase (U L^−1^)	7.00–55.00	52.72	—	71.13
Aspartate aminotransferase (U L^−1^)	8.00–48.00	34.92	531.08	61.44
Gamma-glutamyltransferase (U L^−1^)	8.00–61.00	128.14	190.07	—
Erythrocyte count (×10^6^ μl^−1^)	4.35–6.65	4.2	4.08	3.67
Leucocyte count (×10^3^ μl^−1^)	3.4–9.6	3.2	1.5	1.6
Lymphocyte count (×10^3^ μl^−1^)	0.9–8.0	0.6	0.5	0.5
Neutrophil count (×10^3^ μl^−1^)	1.6–8.0	2.1	0.7	0.7
Platelet count (×10^3^ μl^−1^)	135–317	163	174	128
List of co-administered drugs		1	1, 2, 3, 4, 5, 6, 7	1, 2, 3, 4, 5, 6, 8, 9, 10

(1) isoniazid + rifampicin; (2) amphotericin B lipid complex; (3) fluconazole; (4) enoxaparin sodium; (5) dipyrone; (6) omeprazole; (7) vitamin B_6_; (8) dexchlorpheniramine; (9) sulfamethoxazole + trimethoprim; (10) nicotine transdermal patch.

### 2.7 Pharmacokinetics Analysis

The pharmacokinetic parameters were calculated with Phoenix WinNonlin™, version 8.3.3.33 (Certara USA, Inc., Princeton, NJ, United States) based on the plasma concentration versus time curve. AmB was assessed by non-compartmental analysis (NCA) with the linear trapezoidal linear interpolation method. Unbound fractions (F_u_) were analyzed on the last five samples from the pharmacokinetics curve of each phase as well as on the plasma samples obtained simultaneously as CSF samples.

The amount of AmB excreted (A_e_) in each spontaneous micturition event was calculated by multiplying the urinary concentrations (C_u_) by the respective volume of urine collected (V_u_). The amount excreted in 24 h was obtained by the sum of A_e_ obtained from each interval. The fraction of the dose excreted into the urine (F_el_) was calculated by dividing A_e_ by the dose (Ritschel and Kearns, 1999; Benet, 2010). The renal clearance (CL_ren_) was calculated by multiplying the total clearance (CL) by the fraction of the dose excreted into the urine in 24 h (F_el_). The hepatic clearance (CL_hep_) was calculated by subtracting the renal clearance (CL_ren_) from the total clearance (CL) (Benet, 2010).

## 3 Results

**FIGURE 2:**
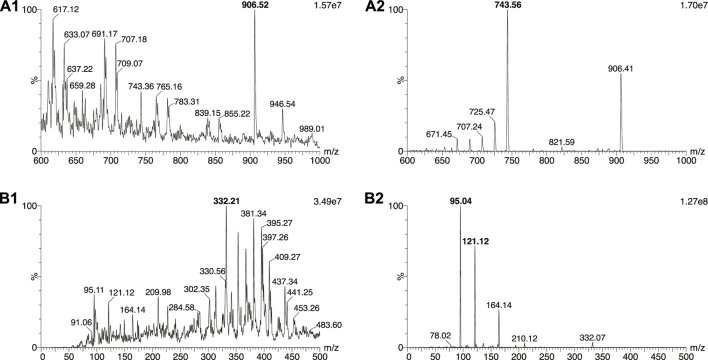
Mass spectra of **(A1)** amphotericin B and **(B1)** internal standard piroxicam, and their respective product ions **(A2, B2)**.

**FIGURE 3:**
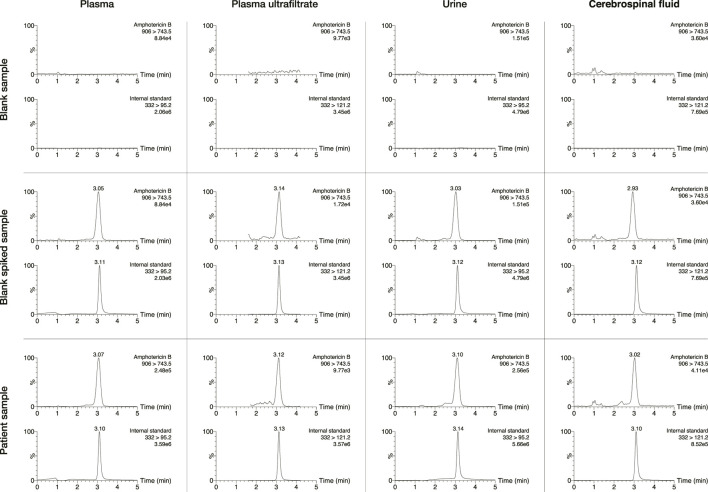
Chromatograms of blank, spiked, and patient’s samples for human plasma, plasma ultrafiltrate, urine, and cerebrospinal fluid. The patient’s samples were enriched with the internal standard piroxicam.

**FIGURE 4:**
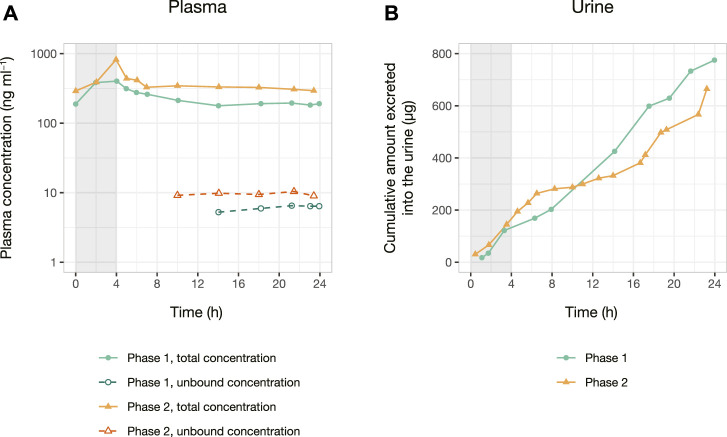
Pharmacokinetics data of a patient treated for neurocryptococcosis with 100 mg of amphotericin B lipid complex as a 4 h i. v. infusion. Total and unbound plasma concentration versus time curve **(A)** is presented in ng ml^−1^, and the cumulative amount excreted into the urine versus time curve **(B)** is presented in μg. Phase 1 was conducted on the 4th day of administration of amphotericin B, whereas phase 2 was conducted on the 16th day of treatment with the drug. The gray area indicates the length of the i. v. infusion (4 h) in both phases.

**FIGURE 5:**
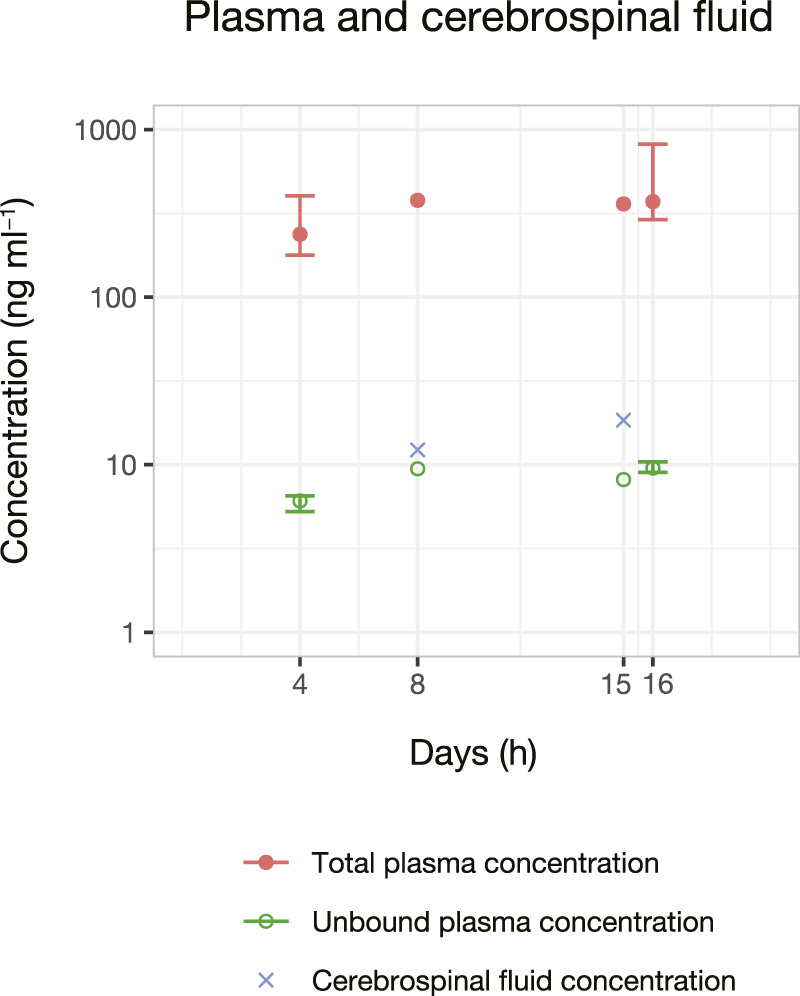
Amphotericin B concentrations in plasma and cerebrospinal fluid samples versus time. Plasma samples are expressed as total and unbound concentrations. Bars (when present) represent the maximum and minimum concentrations observed during a 24 h pharmacokinetic serial sample. Unbound concentration determined in the last five plasma samples collected at the terminal elimination time as described in Figure 1.

The initial experiments of AmB analysis in plasma and urine were performed using the Micromass Quattro Micro™ triple quadrupole mass spectrometer (Waters Corporation, Milford, MA, United States). However, the equipment presented a lower sensitivity considering the expected AmB concentrations in biological samples. Thus, the methods were development using the system XEVO TQ-S^®^ triple quadrupole mass spectrometer (Waters Corp., Milford, MA, United States) previously described. Figure 2 shows the protonated ([M + H]^+^) precursor ions and their respective product ions. AmB was monitored in the transition m/z 906 → 743. The IS piroxicam was chosen in the absence of a commercially available deuterated standard for AmB. Piroxicam was a suitable IS for a sample preparation method applying protein precipitation and presented retention times similar to AmB (differences of 0.10–0.19 min depending on the biological matrix, Figure 3). The sequential analysis in plasma, plasma ultrafiltrate, urine, and CSF are presented in Figure 3. The methods for quantifying of AmB in the four biological matrices were applied to the investigation of the pharmacokinetics of AmB in a male patient treated for neurocryptococcosis. The patient’s anthropometric, biochemical, and hematological parameters, as well as a complete list of medications in use by the time of phases 1 and 2, are presented in Table 1. The validation parameters for the methods of quantification of AmB in plasma, plasma ultrafiltrate, urine, and CSF can be found in Table 2. All validation parameters relative errors were within the range of ±15% and coefficients of variation below 1%. The pharmacokinetic profiles of AmB in each phase of the study are shown in Figure 4 for both plasma and urine. The unbound concentrations of AmB in plasma were determined in the last five samples comprising the terminal elimination phase and are presented in Figure 4 and Table 3, with mean values of 3.25 (±0.22) % in phase 1 and 2.99 (±0.27) % in phase 2. Two CSF samples were obtained in the time between phases 1 and 2. The concentration of AmB in these samples and the relationships between the concentration in CSF and plasma as total and unbound concentrations collected simultaneously are described in Table 4. The ratios of total plasma concentration by CSF concentration ratios were 30.90 and 19.53, respectively at the 8th and 15th days of treatment with AmB. In contrast, the ratios of unbound plasma concentration by CSF concentration were 0.77 and 0.44, respectively at the 8th and 15th days, demonstrating the increasing concentrations of AmB in the CSF throughout the treatment. An overview of AmB concentrations determined in plasma as total and unbound concentrations and in CSF are shown in a comprehensive timeline in Figure 5. Table 5 presents the pharmacokinetic parameters of AmB, the amount excreted into the urine in 24 h, and the renal and hepatic clearances. All datasets generated for this study are included in the article’s Supplementary Material.

**TABLE 2:** Validation for the methods of quantification of amphotericin B in plasma, plasma ultrafiltrate, urine, and cerebrospinal fluid.

	Plasma		Plasma ultrafiltrate		Urine		Cerebrospinal fluid
**Matrix Effect**								
Matrix factor normalized by IS (CV, %)	1.58 (8%)		1.41 (13%)		1.74 (9%)		1.14 (9%)	
**Linearity (ng ml^−1^)**	5–1,000		0.625–250		5–1,000		0.1–250	
Linear equation	y = 0.00121752⋅x + 0.00369099		y = 0.271171⋅x−0.0166566		y = 0.00139923⋅x+0.0026258 0.997705		y = 0.058589⋅x + 0.000938927	
*r* ^*2*^	0.996708		0.981444		0.997705		0.992380	
**Non-specific binding to the membrane**			**CV**	**RE**				
LCQC	—		11	−14	—		—	
HCQC	—		8	15	—		—	
**Precision (CV, %) and Accuracy (RE, %)**	**CV**	**RE**	**CV**	**RE**	**CV**	**RE**	**CV**	**RE**
Within-run								
LLOQQC	7	5	5	5	7	−1	5	7
LCQC	2	1	6	11	3	−2	1	6
MCQC	2	0	2	−6	3	1	2	−4
HCQC	2	−1	13	2	4	−5	1	−2
DCQ (plasma 1:4, v/v; plasma ultrafiltrate 1:10, v/v; urine 1:4, v/v)	2	−4	4	−10	3	6	—	—
Between-run								
LLOQQC	9	−1	13	−1	7	3	7	0
LCQC	5	2	8	12	5	0	2	6
MCQC	6	0	11	1	4	4	3	−2
HCQC	4	−2	10	0	7	−4	5	−8
DCQ (plasma 1:4, v/v; plasma ultrafiltrate 1:10, v/v; urine 1:4, v/v)	5	1	3	−9	4	6	—	—
**Stabilities**	**CV**	**RE**	**CV**	**RE**	**CV**	**RE**	**CV**	**RE**
Freeze and thaw	*−*70°C, 25°C		—		−70°C, 25°C		−70°C, 25°C	
LCQC	6	−9	—	—	5	−6	5	4
HCQC	4	−12	—	—	3	2	4	8
Short-term temperature	25°C, 4 h		25°C, 2 h		25°C, 4 h		4 °C, 2 h	
LCQC	2	6	6	0	5	−6	3	2
HCQC	3	2	3	−6	6	−1	1	−2
Post-preparative	15°C, 24 h		12°C, 20 h		15°C, 24 h		15°C, 24 h	
LCQC	6	−2	5	8	7	−1	3	−3
HCQC	2	−11	6	3	3	−2	2	−9

IS: internal standard; CV: coefficient of variation, expressed as percentage; RE: relative error (inaccuracy) expressed as percentage; LLOQQC: lowest limit of quantification quality control; LCQC: low concentration quality control, MCQC: medium concentration quality control; HCQC: high concentration quality control; DQC: dilution quality control.

**TABLE 3:** Unbound fraction of amphotericin B in plasma. The unbound concentration was determined in the last five plasma samples comprising the terminal elimination phase.

Phase 1 (4th day)	14 h	18 h	21 h	23 h	24 h	Mean (%)	SD	CV (%)
Total concentration (ng ml^−1^)	178.03	190.92	194.72	182.28	190.74			
Unbound concentration (ng ml^−1^)	5.25	5.94	6.51	6.40	6.40			
F_u_ (%)	2.95	3.11	3.34	3.51	3.36	3.25	0.22	6.83
**Phase 2 (16th day)**	**10 h**	**14 h**	**18 h**	**21 h**	**23 h**	**Mean (%)**	**SD**	**CV (%)**
Total concentration (ng ml^−1^)	344.72	331.94	326.86	306.94	293.45			
Unbound concentration (ng ml^−1^)	9.16	9.82	9.43	10.40	9.01			
F_u_ (%)	2.66	2.96	2.89	3.39	3.07	2.99	0.27	8.97

F_u_: unbound fraction in plasma.

**TABLE 4:** Total and unbound concentrations of amphotericin B in plasma and in cerebrospinal fluid samples.

	8th day	15th day
Total plasma concentration (ng ml^−1^)	378.83	360.25
Unbound plasma concentration (ng ml^−1^)	9.46	8.15
F_u_ (%)	2.50	2.26
CSF concentration (ng ml^−1^)	12.26	18.45
Total plasma concentration/CSF concentration ratio	30.90	19.53
Unbound plasma concentration/CSF concentration ratio	0.77	0.44

F_u_: unbound fraction in plasma. CSF: cerebrospinal fluid.

**TABLE 5:** Pharmacokinetics parameters of amphotericin B in a patient with neurocryptococcosis at the beginning and at the end of the treatment.

	Phase 1	Phase 2
Amphotericin B dose (mg)	100	100
Amphotericin B dose (mg kg^−1^)	1.85	1.85
C_max_ (ng ml^−1^)	402.43	820.02
t_½__λz_ (h)	29.89	35.07
λ_z_ (h^−1^)	0.023	0.020
AUC_0–24_ (h ng ml^−1^)	5642.79	8671.01
AUMC_0–24_ (h^2^ ng ml^−1^)	58,789.75	92,773.66
MRT (h)	8.42	8.70
A_e_ (μg)	775.17	664.70
F_el_ (%)	0.008	0.007
V_ss_ (L)	302.94	206.89
V_ss_ (L kg^−1^)	5.59	3.67
CL (L h^−1^)	7.21	4.25
CL (L h^−1^ kg^−1^)	0.133	0.076
CL_ren_ (L h^−1^)	0.056	0.028
CL_ren_ (L h^−1^ kg^−1^)	0.00103	0.00050
CL_hep_ (L h^−1^)	7.15	4.22
CL_hep_ (L h^−1^ kg^−1^)	0.077	0.047

C_max_, maximum observed concentration; t_½__λz_, terminal elimination half-life; λ_z_, terminal elimination rate constant; AUC_0–24_, area under the plasma concentration versus time curve, from time zero to 24 h; AUMC_0–24_, area under the first moment of the plasma concentration versus time curve, from time zero to 24 h; MRT, mean residence time; A_e_, amount of the dose recovered in the urine; F_el_, fraction of the dose excreted into the urine; V_ss_, volume of distribution in the steady state; CL, total clearance; CL_ren_, renal clearance; CL_hep_, hepatic clearance.

## 4 Discussion

The present study aimed to develop a method capable of quantifying AmB in CSF at any given time of the treatment in addition to plasma, plasma ultrafiltrate, and urine. The quantification of AmB in CSF has been a methodological challenge from the last decades to the present day, with studies reporting a large number of CSF samples in which AmB concentrations are undetectable or below the LLOQ in a fraction of or all samples, despite the observation of clinical efficacy (Leenders et al., 1997; Wurthwein et al., 2005; Hamill et al., 2010; Strenger et al., 2014). Thus, the method developed for quantification of AmB in human CSF combines the convenience of a smaller sample volume (100 μl) and lower LLOQ (0.1 ng ml^−1^) compared to those reported in the literature, with sample volumes of 1,000 μl (Liu et al., 1995), 500 μl (Strenger et al., 2014), and 250 μl (Wirth et al., 2018) and LLOQ’s of 500 ng ml^−1^ (Strenger et al., 2014), 1 ng ml^−1^ (Liu et al., 1995), and 0.5 ng ml^−1^ (Xiong et al., 2009).

The development of the chromatographic method was initially carried out following the method described by Su et al. (2018), which uses an Ascentis C18 reverse-phase column, 5 μm particles, 50 × 4.6 mm and applies a gradient of 0.2% formic acid and 5 mM ammonium acetate in water (solution A) and 0.2% formic acid in acetonitrile (solution B). During the parameters optimization step with an infusion of AmB, it was observed in our experiments that only 0.2% formic acid contributed to the increase in the signal of the analyte. The initial tests were performed using the reversed-phases columns Purospher RP-18e (123 × 3 mm), RP-Select B (125 × 4.6 mm), and RP-Select B (250 × 4.6 mm), all with 5 µm particles maintained at 40°C and protected by a guard column of the same specification. The columns were tested in different mobile phase combinations containing 0.1 and 0.2% formic acid in both water and acetonitrile in the proportions 70:30, 60:40, 50:50, 40:60 and 30:70 (v/v). The best chromatographic profile for the analysis of AmB in plasma and urine samples was observed in the RP-Select B reverse phase column (125 × 4.6 mm) with a mobile phase consisting of a mixture of 0.1% formic acid in water and 0.1% formic acid in acetonitrile in a 40:60 (v/v) ratio. However, analysis of AmB in CSF and plasma ultrafiltrate samples required changing the proportion of mobile phase constituents (50:50, v/v) to obtain better shaped and more symmetrical chromatographic peaks (Figure 3). Initial tests were performed with the column maintained at 40°C (Su et al., 2018), but considering that at 25°C the chromatographic profile remains constant, the lowest temperature was chosen.

Considering the absence of a commercially available deuterated standard for AmB, the search for an IS was based on previously published methods. Piroxicam, used in previous studies with spectrophotometric detection, proved to also be adequate for the protein precipitation process and mass spectrometry detection used in the present study (Campanero et al., 1997; Echevarría et al., 1998; Hope et al., 2012). It is noteworthy that piroxicam was monitored at transition m/z 332 → 95 in the analysis in plasma and urine, and at transition m/z 332 → 121 in the ana analysis in plasma ultrafiltrate and CSF to avoid interference with matrices components (Figure 3).

The methods for the chromatographic analysis of AmB in biological matrices use the solid-phase extraction technique, which makes the analysis of a large number of samples more expensive (Liu et al., 1995; Lee et al., 2001; Bekersky et al., 2002b; Bellmann et al., 2003; Su et al., 2018; Van Daele et al., 2021). Sample preparation was initially tested with the intention of replacing solid-phase extraction by protein precipitation, aiming for lower costs of analysis and shorter times of sample preparations, key aspects of pharmacokinetic studies in which large numbers of samples are analyzed using sensitive methods. The best result was obtained by precipitating plasma, plasma ultrafiltrate, urine, and CSF samples with a 0.1% formic acid solution in acetonitrile, followed by dilution of the respective supernatants with a 0.1% formic acid solution in water.

The preparation of plasma, plasma ultrafiltrate, urine, and CSF samples with protein precipitation followed by dilution of the supernatants resulted in the absence of any significant matrix effect, evaluated as the observation of the coefficients of variation of matrix factor values normalized by the IS less than 10% (Table 2).

The linearity of the methods for the analysis of the concentration of AmB in plasma (5–1,000 ng ml^−1^), plasma ultrafiltrate (0.6–250 ng ml^−1^), urine (5–1,000 ng ml^−1^), and in CSF (0.1–250 ng ml^−1^, Table 2) proved to be adequate for the application in clinical pharmacokinetics studies, with determination coefficients (*r* ^2^) greater than 0.98 for plasma ultrafiltrate and 0.99 for the other matrices. The wide linear range will enable the quantification of AmB in biological fluids from patients treated with different AmB formulations, such as deoxycholate or lipid formulations (Ayestarán et al., 1996). The upper limit of quantification (ULOQ) values of 1,000 ng ml^−1^of plasma or urine and 250 ng ml^−1^ of plasma ultrafiltrate and CSF were the highest within the linear ranges for which no carryover effect was observed. The methods developed and validated in this study using aliquots of 25 μl of human plasma or urine and 100 μl of human plasma ultrafiltrate and CSF, with LLOQ values of 5 ng ml^−1^ for plasma or urine, 0.6 ng ml^−1^ for plasma ultrafiltrate, and 0.1 ng ml^−1^ for CSF can be considered the most sensitive ones described so far. Overall, the methods described here are 3.2–1,500 times more sensitive than the LC-MS/MS methods previously described in the literature (Lee et al., 2001; Bekersky et al., 2002a; Xiong et al., 2009; Deshpande et al., 2010; Qin et al., 2012; Al-Quadeib et al., 2014; Strenger et al., 2014; Su et al., 2018; Van Daele et al., 2021). Despite reaching the highest sensitivity in the literature, the methods could be further adjusted to use even lower sample volumes by adding a final concentration step, in which the supernatants are evaporated and reconstituted in lower volumes. Even though undesirable for clinical pharmacokinetic studies, since it adds a new time-consuming step, this modification would allow for the methods to be applied to animal models, such as mice.

The methods of analysis of total concentrations of AmB in plasma, urine, and CSF and the unbound concentration in plasma proved to be precise and accurate, with coefficients of variation and relative error values below 12% (Table 2). The freeze and thaw, short-term temperature, and post-preparative stabilities studies showed coefficients of variation and relative error values equal to or less than 12% when quantified with freshly prepared calibration curves (Table 2).

AmB is a highly lipophilic drug administered parenterally due to its low oral absorption. The administration of AmB in lipid formulation results in faster AmB accumulation in peripheral tissues, in lower plasma concentrations, and minimizes adverse reactions compared to the deoxycholate formulation (Ayestarán et al., 1996). The methods validated in the present study were applied to investigate the pharmacokinetics of AmB following infusions of 100 mg every 24 h for 16 days administered as a lipid complex throughout the treatment of a neurocryptococcosis patient.

All collected biological samples (Figure 1) were quantified considering the high sensitivity of the method. It is important to highlight that AmB was successfully quantified in CSF samples collected on the 8th and 15th days of the treatment (Figure 5).

Clinical pharmacokinetics of AmB lipid complex was previously reported in healthy volunteers (Kan et al., 1991) and in patients with systemic fungal infections (Adedoyin et al., 2000). The AmB pharmacokinetic parameters such as total clearance and volume of distribution at steady state are dose-dependent considering that the drug accumulates in the tissues. The cited authors observed total clearance values in healthy volunteers ranging from 0.07 to 0.09 L h^−1^ kg^−1^, values similar to those presented in Table 5 for the patient with neurocryptococcosis (0.133 L h^−1^ kg^−1^ on the 8th day to 0.076 L h^−1^ kg^−1^ on the 15th day). However, the administration of AmB lipid complex at 5 mg kg^−1^ day^−1^ to patients with fungal infections resulted in higher total clearance values (17.8 ± 5.2 L h^−1^) compared to the values presented in Table 5 for the investigated patient with neurocryptococcosis treated with daily infusions of 100 mg (7.21 L h^−1^ on the 8th day to 4.25 L h^−1^ on the 15th day). However, when the different studies are dose-normalized, all the values are equivalent. Considering that in the present study AmB was also quantified in urine, it was possible to verify that total clearance values are very similar to hepatic clearance (0.077 L h^−1^ kg^−1^ on the 8th day and 0.047 L h^−1^ kg^−1^ on the 15th day) due to its elimination mainly by biliary secretion; Table 5.

AmB lipid complex is highly distributed. Volumes of distribution at steady state were also evaluated in healthy volunteers (1.7–3.9 L kg^−1^) (Kan et al., 1991) and in patients with systemic fungal infections (865 ± 347 L) (Adedoyin et al., 2000). Volumes of distribution at steady state for the investigated patient were 5.59 (8th day) to 3.67 L kg^−1^ (15th day) (Table 5); values close to the cited studies when dose-normalized.

AmB unbound fraction following the lipid formulation was evaluated in the investigated neurocryptococcosis patient. The data reported in Tables 3 and 4 show AmB unbound fraction values of 3.25 (4th day), 2.50 (8th day), 2.26 (15th day), and 2.99 (16th day). Although there are no reports in the literature about AmB unbound fraction following the lipid formulation, the administration of other formulations such as liposomal or deoxycholate also result in unbound fraction values of 4.5 and 20.6%, respectively (Bekersky et al., 2002b).

In the present study, total plasma concentration/CSF concentration ratios ranged from 30.90 (8th day) to 19.53 (15th day), whereas unbound plasma concentration/CSF concentration ratio ranged from 0.77 (8th day) to 0.44 (15th day), showing increasing concentrations of AmB in the CSF with the evolution of the treatment. Strenger et al. (2014) reported total plasma concentration/CSF concentration ratios ranging from 124 to 2,391 in haemato-oncological pediatric patients treated with liposomal AmB (3 mg kg^−1^ day^−1^) in samples evaluated from 0 to 48 h after drug infusion. However, total plasma concentration/CSF concentration and unbound plasma concentration/CSF concentration ratios were not found in the available literature following the administration of lipid formulation.

In conclusion, the present study described the most complete and sensitive methods for AmB analysis in plasma, plasma ultrafiltrate, urine, and CSF applied to a clinical pharmacokinetic study following the administration of the drug as a lipid complex in one patient with neurocryptococcosis. Previous clinical studies failed to fully assess all biological matrices due to low sensitivity and higher matrices volumes, especially for CSF. The method can be applied to investigate the pharmacokinetics of AmB in CSF at any given time of the treatment.

## Data Availability

The original contributions presented in the study are included in the article/Supplementary Material, further inquiries can be directed to the corresponding author.
